# Neuropsychological Sequelae, Quality of Life and Adaptive Behavior in Children and Adolescents with Anti-NMDAR Encephalitis: A Narrative Review

**DOI:** 10.3390/brainsci11111387

**Published:** 2021-10-22

**Authors:** Samuela Tarantino, Roberto Averna, Claudia Ruscitto, Fabiana Ursitti, Michela Ada Noris Ferilli, Romina Moavero, Laura Papetti, Martina Proietti Checchi, Giorgia Sforza, Martina Balestri, Teresa Grimaldi Capitello, Federico Vigevano, Stefano Vicari, Massimiliano Valeriani

**Affiliations:** 1 Unit of Clinical Psychology, Bambino Gesù Children’s Hospital, IRCCS, 00165 Rome, Italy; martina.proietti@opbg.net (M.P.C.); teresa.grimaldi@opbg.net (T.G.C.); 2 Child and Adolescence Neuropsychiatry Unit, Bambino Gesù Children’s Hospital, IRCCS, 00165 Rome, Italy; roberto.averna@opbg.net (R.A.); stefano.vicari@opbg.net (S.V.); 3 Child Neurology and Psychiatry Unit, Tor Vergata University of Rome, 00133 Rome, Italy; claudia.ruscitto@opbg.net (C.R.); romina.moavero@opbg.net (R.M.); 4 Department of Neurology, Bambino Gesù Children’s Hospital, IRCCS, 00165 Rome, Italy; fabiana.ursitti@opbg.net (F.U.); michela.ferilli@opbg.net (M.A.N.F.); laura.papetti@opbg.net (L.P.); giorgia.sforza@opbg.net (G.S.); martina.balestri@opbg.net (M.B.); federico.vigevano@opbg.net (F.V.); massimiliano.valeriani@opbg.net (M.V.); 5 Center for Sensory-Motor Interaction, Denmark Neurology Unit, Aalborg University, 9100 Aalborg, Denmark

**Keywords:** anti-NMDAR, children, adolescents, neuropsychology, adaptive behavior

## Abstract

Anti-*N*-methyl-d-aspartate receptor (anti-NMDAR) encephalitis is a rare autoimmune illness characterized by a constellation of often severe, but treatable, psychiatric and neurological symptoms. Whereas symptoms such as psychosis and bizarre and abnormal motor behavior are common in adults, pediatric patients typically present with behavioral changes, irritability and sleep dysfunction. The recovery phase is usually slow and may be associated with longstanding adaptive, behavioral and neuropsychological problems. Very few studies explored the cognitive and adaptive sequelae in children with anti-NMDAR encephalitis. The present review article suggests that, although most children and adolescents return to their daily life and previous activities, they may have a low quality of life and show neuropsychological sequelae involving language, memory, especially verbal memory, and attentional resources, even after several months from the hospital discharge. In particular, the available results reveal difficulties in cognitive skills involving executive functions. This impairment is considered the “core” of the cognitive profile of young patients with anti-NMDAR encephalitis. On the other hand, some cognitive skills, such as general intelligence, show good overall recovery over time. Additional neuropsychological research evaluating larger samples, more homogenous methods and longitudinal studies is required.

## 1. Introduction 

First described in 2007, anti-*N*-methyl-d-aspartate receptor (anti-NMDAR) encephalitis is a rare autoimmune illness [1]. It is characterized by a constellation of often severe, but treatable, psychiatric and neurological symptoms related to the presence, in cerebrospinal fluid, of antibodies against the GluN1 subunit of the NMDA receptor [2,3,4]. Although initially identified as a paraneoplastic disease in young women with ovarian teratoma [1,5,6], anti-NMDAR encephalitis has very often an unknown etiology, especially in pediatric age [7,8,9]. This disease has been recognized in patients of all ages and spans a broad age range (4 months–85 years) [2,3,8]. Epidemiological data show that this disease is one of the most common causes of encephalitis in pediatric age and it is second only to acute demyelinating encephalomyelitis (ADEM) [10]. Although the clinical picture, course and evolution are similar to those of adults, symptoms can vary substantially in pediatric age [2,11,12,13]. The symptoms of anti-NMDAR encephalitis usually evolve through stages [1,13,14]. Prodromal symptoms are described in both adult (85%) and pediatric age (from 32% to 51%) [8,11,15,16]. This phase is often characterized by “cold-like symptoms” such as fever, headache, vomiting, nausea, myalgias, diarrhea or upper respiratory-tract symptoms [7,8,13,17]. Within a few days (usually no more than 2 weeks), most patients advance into a phase characterized by a combination of psychiatric features (such as anxiety, agitation, bizarre behavior, delusional paranoid thoughts and hallucinations) and neurological decline [1,7,18,19]. Whereas psychosis and bizarre and abnormal motor behavior (including catatonia) are common in adults (70–90%), neurological symptoms occur more often in children, especially when aged below 12 years (60%) [2,3,7,11]. In adolescents, the disease may present with a combination of both psychotic and neurological symptoms [2]. Children typically present behavioral changes, irritability, aggressive behavior, manic symptoms, behavioral outbursts, sleep dysfunction, anxiety and hyperactivity [11,20,21,22,23]. The main neurological symptoms of pediatric anti-NMDAR encephalitis are movement disorders (such as dyskinesias, choreoathetosis, tremor and dystonia), seizures and insomnia [8,11,13,24]. Autonomic dysfunctions, including abnormal heart and respiratory rates and blood pressure modifications, are common in both adults and children (in about 50% of adolescents and 40% of preadolescent patients) [7,11,22]. Early recognition of symptoms and identification of NMDAR antibodies allow clinicians a prompt pharmacological intervention and often a better outcome. Corticosteroids, plasmapheresis and immunoglobulins are the first-line treatments [25]; however, second-line immunotherapy (cyclophosphamide and/or rituximab) is very often required (for almost 50% of pediatric patients) [2,3,26]. Despite the severity of symptoms and course of the disease, most patients (75–85%) show a favorable outcome [2,7,27]. The recovery phase is usually slow and can take from months to years [2]. As for several chronic diseases, this process may be associated with longstanding adaptive, behavioral, and social problems that may require rehabilitative treatment and extensive psychosocial care [20,26]. Increasingly, the data on adults with anti-NMDAR encephalitis show substantial long-term cognitive impairment, sometimes up to several years after clinical recovery, with a potential negative impact on work performance and quality of life [28,29,30]. In particular, protracted major neuropsychological sequelae in adults include memory deficits (especially verbal memory and working/short memory), attentional and processing speed difficulties and executive dysfunctions [28,30]. With regard to children and adolescents, data concerning this subject are far fewer and have never been reviewed.

### 1.1. Aims

In this review article, we aimed to focus more attention to pediatric anti-NMDAR encephalitis outcomes. We will investigate the current literature on neuropsychological impairment of children and adolescents who have suffered from this disease, attempting to describe their long-term cognitive profile. Moreover, in order to better understand patients’ rehabilitative needs, we explore the current literature on quality of life and adaptive behavior of pediatric patients with a clinical history of anti-NMDAR encephalitis.

### 1.2. Hypothesis

**Hypothesis** **1.** *Given the results issued by studies based on adult patients, we hypothesize that long-term neuropsychological sequelae may also be common at pediatric age, particularly involving memory along with executive functions. Furthermore, we expect patients to have a low quality of life and adaptive behavior difficulties.*

## 2. Methods

Appropriate studies were identified using Web of Science and MEDLINE. Given that anti-NMDAR was only officially categorized in 2007, we considered studies published from the period of 2007 to April 2021. Our search was limited only to the English language.

**Figure 1:**
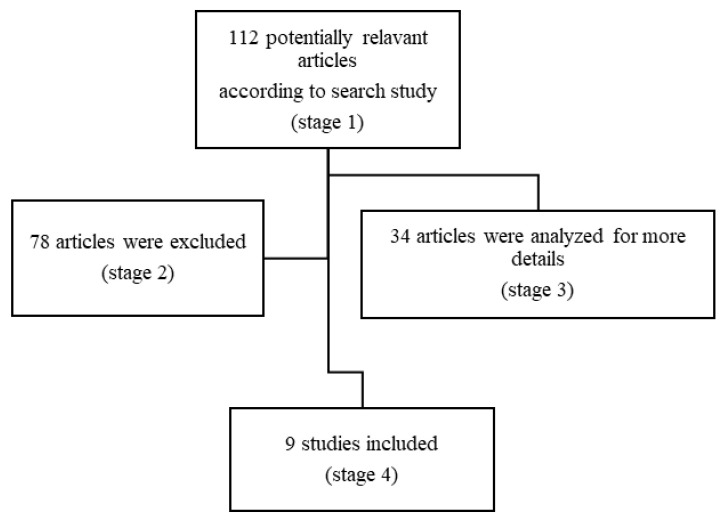
Flow diagram of the study methodology.

Search words were “pediatric” or “children”, “anti-NMDAR encephalitis” or “pediatric anti-N-Metil-D-Aspartate Receptor encephalitis” and “outcome”, “cognitive outcome”, “neuropsychology”, “adaptive behavior” and “quality of life”. Our search focused on the age group ranging from 0 to 18 years, although we also considered articles that evaluated adults but also included patients under the age of 18 years. The filters included reviews and articles, retrospective studies (RS), multicenter studies and clinical trials (CT). Figure 1 shows the search process.

## 3. Results

Using the above described strategy, we identified 9 eligible articles. Among them, 5 were case studies (2 single case study, 3 with multiple), 1 was a retrospective study and 3 original articles (in one of them, the collected data were evaluated retrospectively) (Figure 1). All neuropsychological test results were compared with normative data; no study had a matched control group.

Moreover, several different tests were employed to measure neuropsychological abilities across studies.

### 3.1. General Intelligence

Research on adults evidenced a good outcome of overall intellectual performance. Similar results have been found in both patients with anti-NMDAR encephalitis and healthy subjects [30]. Data investigating the possible impact of this disease on general intelligence in pediatric patients is sparse [21,31,32]; in particular, there is a lack of studies evaluating the profile of children’s intellectual performance in detail. However, with regard to general intelligence, few studies and case reports available on children/adolescents have described a positive outcome over time. In 2010, few years after the original characterization of anti-NMDAR encephalitis, Poloni et al. described the case of an 8-year-old girl diagnosed with Sebire syndrome and whose serum was found positive for anti-NMDA receptor antibodies (see Table 1) [21]. Fifteen months after the onset of symptoms, the patient underwent a detailed cognitive evaluation (Wechsler Intelligence Scale for Children, 4th edition) [33], showing a total IQ of 78, specifically impaired by attention and working memory problems. The authors, however, did not provide a second long-term assessment. Some years later, Matricardi et al. through standardized tests (Wechsler Intelligence and Leiter Scales), [34,35] showed a general improvement in intellectual abilities in 13 children (10 prospective and 3 retrospective patients) (Table 1) [31]. Prospective patients were initially evaluated after a median of three months from symptoms onset (range 1–12, Time 1) and were subjected to a second evaluation after a median of 27 months from disease onset (range 12–60 months, Time 2). Among nine patients who were able to undergo the assessment within six months from the onset of the disease (Time 1), only four patients had normal general intelligence. One patient, who was examined 12 months after symptom onset, had global cognitive functioning and reasoning in the lower normal range. At Time 2, however, most patients had general intellectual abilities within the normal range. More recently, Caianelli et al., described a good outcome of general intelligence in 5/6 children with anti-NMDAR encephalitis (Table 1) [32]. By using Raven’s Matrices [36], which explore general non-verbal intelligence and reasoning, only one patient performed below the normal range one month after hospital discharge. At the last assessment, performed after a mean of 35 months from symptoms onset (range 24–48 months), even the last patient showed an improvement (from a z score of −2.5 to −1.26). In younger children, psychomotor regression is also a common feature of anti-NMDAR encephalitis [32,37,38]. Although a standardized assessment was not performed to evaluate the impact of the disease on psychomotor development, Ori Scott described an unusual case of autistic regression in a healthy 33-month-old boy [38]. After a mild febrile upper respiratory tract infection, the child developed a progressive regression by becoming non-communicative, losing eye contact and language. As from the third day of treatment (a 5-day course of immunoglobulins), the child showed an improvement in neurological symptoms (movements disorders), social skills and language, which became functional as in the pre-illness stage. These findings are in agreement with those from a report by Iadisernia et al., who described the case of a 5-year-old girl with anti-NMDAR encephalitis showing an improvement from a General Quotient (GQ, evaluated by Griffiths scales) of 85 (twenty-two months after the onset) to a GQ of 96 (one year after the first evaluation) [37,39]. Even if a good prognosis concerning general intelligence is common, there are a few exceptions. In a study by Cainelli et al., a 15-month-old child, serially evaluated over a time interval of 27 months, showed a gradual psychomotor deterioration, particularly affecting language skills, with a GQ below the normal range [32]. There is evidence that late immunotherapy may have a negative influence on general intelligence or psychomotor outcome [31,37]. Mild mental impairment, assessed 24 months after symptom onset, was found in a 4-year-old boy who underwent immunotherapy just three months before the cognitive evaluation [37]. The role of a late pharmacological intervention was also evidenced by Matricardi et al. [31]. Among three patients who were retrospectively recruited and tested in a single assessment (31, 86 and 112 months after disease onset), only one patient, who was tested 112 months after onset, had a general intelligence below the normal range (Wechsler Intelligence Scale, IQ Total of 72) [35]. It is noteworthy that the child was first diagnosed and treated during a relapse, four years after the initial symptoms of the disease (2005) [31].

### 3.2. Language 

During the early stage of the disease, patients may show a decline or alteration in language skills [3,7,16,22,41,42]; long-term follow-ups conducted on adults, however, evidenced a general good outcome in both receptive and expressive abilities [28]. Although changes in language and speech are even more recurrent in children and adolescents with this encephalitis [43], data on the long-term evolution of these functions are sparse, and the results are not conclusive. To the best of our knowledge, only five studies measured the evolution of language in children/adolescents with anti-NMDAR encephalitis [31,32,37,40]. Some reports did not use standardized tests [21] or did not include a second or long-term follow-up [37,40]. Poloni described the case of a 6-year-old girl who was found positive to anti-NMDAR antibodies [21]. Within a week of the onset of symptoms, the patient developed bizarre language and become intermittently mute. Despite an ongoing recovery, the patient was still receiving special assistance because of language, memory and social difficulties after 22 months from symptoms onset. A structured neuropsychological evaluation on language and other cognitive functions, however, was not described by the authors. Matricardi et al. [31], by using a battery of structured tests (the Italian Battery BVN 5–18, the Token Test for Children 2nd edition and the Test for Reception of Grammar) [44,45,46], described difficulties in verbal comprehension, naming, phonemic and semantic verbal fluency in most patients (7/10) who underwent cognitive assessment at Time 1 (1–12 months after symptoms onset) (Table 1). However, an improvement in language abilities (receptive and expressive) was found in most of them at Time 2 (12–60 months after the onset). In two children (respectively, a 4-year-old girl and a 5-year-old boy), Iadisernia et al. described a low performance in receptive lexicon, rapid naming and verbal fluency 10 months and 12 months after the onset of the disease, respectively (Table 1) [37]. However, the patient (4-year-old girl) that was retested after 22 months from the onset showed a good outcome in language (and other abilities), achieving her inferred premorbid status. In 2014, an unusual case of aphasia was described by Deiva [41]. The patient (a 4-year-old girl) showed a good response to treatment (intravenous Rituximab, 375 mg/m^2^, started a month and a half after disease onset) and the resolution of symptoms occurred within a few weeks. In disagreement with these data, subtle deficits and difficulties in language skills were described in an American inter-institutional case series [47]. Despite a gradual improvement in cognitive tasks over the recovery phase, in a structured follow-up conducted from hospitalization to long-term clinical observation (6–24 months after symptoms onset), three adolescents showed fragility in comprehension of instructions, confrontational naming, repetition and verbal fluency. Long-term impairment in naming performance (evaluated by the Boston Naming Test) [48] was also found in a Dutch study, performed on 16 children diagnosed with anti-NMDAR encephalitis (Table 1) [40]. However, patients were tested retrospectively, with very different timing (5–91 months after symptoms onset).

### 3.3. Attention and Executive Functions

Several studies on adults reported difficulties in attention, processing speed and executive functions during both the acute (<12 months from diagnosis) and chronic phases (>12 months) of the disease [28,49,50,51]. Although most children show general improvement in their intellectual abilities and can resume their normal activities, there is evidence that subsyndromal and protracted difficulties in attention skills and executive functions may affect school performances [22,31]. Despite the small body of literature available, protracted dysfunctions in attention and executive functions are expected even after more than 12 months from the onset of symptoms [31,37]. Iadisernia et al. described a neuropsychological profile consistent with persistent impairments in selective and prolonged attention, thinking flexibility and problem-solving tasks in two children who underwent a cognitive assessment (Table 1) [37]. At the follow-up (32 months from symptoms onset), the patient who was retested (Bell Test) [52] showed a significant improvement of selective/prolonged attention and problem-solving tasks. Subtle deficits in the area of executive functioning were also described in three patients reported by Hinkle (2017) [47]. In particular, the authors evidenced some executive functioning difficulties, including problem solving, inhibition, planning and organizational abilities (3.5–12 months after the acute observation). Matricardi et al. reported impairment in attention and executive functions not only in a short time assessment (1–12 months after symptoms onset) but also at the long-term follow-up (12–112 months) [31]. The study evidenced that, among 10 patients who were initially tested (Barrage Task, Bell Test and Coding—Wechsler subtest) [35,52], more than half had selective/sustained attention, processing speed and planning dysfunctions. The later evaluation showed residual difficulties in sustained attention, processing speed and planning in most of the patients. Qualitative difficulties in maintaining attention, associated with a specific neuropsychological profile, were also found by Cainelli et al. [32]. Moreover, although the authors did not describe a typical dysexecutive syndrome, they identified a pattern consistent with executive dysfunction, characterized by planning difficulties, defective flexibility, perseveration and intrusion errors in most patients. In particular, within 1 month after discharge, there was still a high prevalence of attention problems (selective and prolonged; 3/5 patients) and pure executive functions (5/5 patients). At long-term follow-up (24–48 months), attentive difficulties persisted in one patient, while executive functions deficits were found in two patients. Impairment of sustained attention performances and processing speed was also confirmed by De Bruijin in almost all tested patients (median follow-up of 31 months) [40]. The study evidenced that a poor outcome in sustained attention was independent from predictors of good outcome (e.g., treatment delay, age of onset, intensive care unit stay) and follow-up time.

### 3.4. Memory

Memory impairment, in particular verbal memory, is commonly reported in adult patients with anti-NMDAR encephalitis both in the acute and chronic phase [28,51,53]. In spite of these findings, data on memory performance in pediatric age are poor. However, memory deficits have been found in children with anti-NMDAR encephalitis even after some years from symptoms onset [22,31,33,40,54]. Moura et al. presented the case of a 15 years-old girl diagnosed with anti-NMDAR encephalitis who, despite improvement in both the neurological and psychiatric symptoms, still experienced difficulties in verbal memory, concentration and attention almost three years after the onset of the disease [22]. However, the clinical observation was not supported by a structured assessment. Using structured tests (Table 1), deficits in verbal memory were described in Hinkle’s cases, although memory performances were not uniformly impaired [47]. Performance in recognition, working memory and retrieval based working memory was low in 2/3 of the patients in the chronic phase, approximatively after 6–24 months from symptoms onset. Low scores in memory ability were confirmed by De Bruijin, who showed difficulties in long-term verbal memory and weakness in short-term verbal and visual memory [40]. Noteworthy, working memory was not impaired in these patients. On the other hand, some authors described a good evolution of memory skills in children with anti-NMDAR encephalitis. In a study by Matricardi et al., most patients (8/10) showed short-term verbal, but not visuo-spatial, memory difficulties at first assessment; however, over the time, several patients showed improvements and the performances became normal in most of them [31]. Cainelli et al. also showed an improvement in memory abilities in 3/6 patients (Table 1) [32].

### 3.5. Visuo-Perceptive and Visual-Motor Integration

As for the neuropsychological abilities described above, few pediatric studies have analyzed the impact of anti-NMDAR encephalitis on visuospatial abilities. In 2012, Iadisernia showed relatively preserved visuo-perceptive and visuo-constructive abilities in one of the two patients evaluated [37]. On the other hand, at the initial assessment (Visual-Motor Integration test), copying, visuo-perception and motor coordination performances were below the average range in the other patient [55]. However, the authors could not evaluate the neuropsychological outcome over the time. Difficulties in visuo-motor integration were reported by Matricardi (Table 1) in most patients evaluated at Time 1 (Visual-Motor Integration and Rey–Osterrieth Complex Figure tests) [31]. As the follow-up progressed, however, children’s performance showed a normalization (with the exception of two retrospectively recruited patients). A good outcome was also described by Hinkle et al. [47]. In their three clinical case reports, the authors evidenced an impairment of visual-motor construction in one patient, due to her impulsive behavior (acute phase); also in this case, the post-acute and outcome evaluations showed a normal performance. The role of executive functions on visual motor skills was described by Caianelli [32]. In the study, all patients had a low performance in visual motor abilities involving executive functions. The long-term follow-up showed deficits in these abilities in 2/4 patients (Table 1).

### 3.6. Quality of Life and Adaptive Behavior

Anti-NMDAR encephalitis sequelae may affect children’s life and their participation in curricular and social activities [22,31,32,40]. These patients may need supports at school and home, together with long-lasting rehabilitation [22,26]. A study by Li et al. revealed that, compared with different types of autoimmune encephalitis, the total direct cost in terms of economic, social and psychological impact of pediatric anti-NMDAR encephalitis may be higher than other autoimmune encephalitis [56]. The involvement of the physical but also psychological and emotional sphere is indeed crucial to understand the burden that this disease can have on the families of both adult and pediatric affected patients. There is evidence that, in adult patients, an inappropriate treatment path could significantly influence the caregiver’s burden [57]. In a recent study, concerns on at least one measure of neuropsychological (executive functioning), adaptive and emotional behavior were reported in almost 90% of caregivers [54].

So far, adaptive behavior and quality of life in patients with anti-NMDAR are still unexplored. To assess the degree of disability and the impact on daily life in patients with anti-NMDAR encephalitis, several studies used tools created for neurological injuries (i.e., the modified Rankin Scale, a stroke disability scales) [58]. These tools, however, may have not adequately captured patients’ perception of quality of life and adaptive behavior. Although not focusing only on anti-NMDAR encephalitis, a study by Ramanuj [59] evidenced the adverse effect of encephalitis (infective, immune-mediated and of unknown etiology) on quality of life, evaluated by the Short Form Health 36 and 10 items surveys [60]. In both pediatric and adult patients, a low score on the Glasgow Outcome Scale (used to investigate the outcome in head injury patients) [61] was strongly associated with poor quality of life [59]. In a recent study based on adults and children with anti-NMDAR encephalitis, Zeng et al. showed that patients’ quality of life, evaluated by the Modified Rankin Scale, was better at relapse than at the onset [62]. Moreover, they found a positive influence of active immunotherapy on quality of life both at the onset and at the relapse.

All these studies did not explore the possible correlation between quality of life and neuropsychological outcome. Focusing on pediatric age, de Bruijin et al. demonstrated that, despite a good functional recovery, patients reported fatigue and poor quality of life [40]. Of note, in this study, the low quality of life was not affected by cognitive dysfunction, such as memory and attention impairments, but only by fatigue. Moreover, as for sustained attention and fatigue, quality of life was not affected by predictors of favorable outcome. The authors hypothesized that children’s reduced awareness of personal difficulties, the general nature of the questionnaire and patients’ adaptation to their new situation could have influenced their results [40]. To the best of our knowledge, only one study evaluated the adaptive behavior in children/adolescents recovered from anti-NMDAR encephalitis. In 2017, Gordon-Lipkin et al. investigated long-term adaptive function and neurologic disability in children and adults with anti-NMDAR encephalitis. Despite no differences being found in neurological disability, children showed worse adaptive behavior outcomes compared with adults [63]. Furthermore, the authors demonstrated that adaptive functions were not affected by cognitive and functional deficits in adult patients. They supposed that an inflammatory disease process, when it occurs in a developing brain, may have more negative consequences, leading to a more severe functional and cognitive outcome [64].

## 4. Discussion

Data on the neuropsychological sequelae, quality of life, and adaptive behavior in patients diagnosed with anti-NMDAR encephalitis are still sparse, especially at pediatric age. Emerging research on children and adolescents, however, provided important insights on their cognitive profile and long-term difficulties. The present review article suggests that, although some cognitive functions, such as general intelligence, may show a progressive decline in the acute phase of the disease, data on long-term follow-up showed a general good recovery over time, especially when early therapy is started [31,32,37]. On the other hand, despite most children and adolescents returning to their daily life and previous activities, they may report fatigue and a low quality of life and have to cope with altered neuropsychological functioning involving language, executive functions, attentional resources and memory, particularly verbal memory, even after several months from the hospital discharge [31,32,37,40]. Although data about the disease financial burden and employment, due to the cognitive decline, are lacking, it is conceivable to expect a great impact also on these aspects. The reviewed literature suggests that cognitive recovery is slow, and several neuropsychological functions may progress with a different and non-linear evolution. Compared with adult age, findings on pediatric case reports and samples appear to be slightly varied and less homogeneous. However, as in adults, available results reveal difficulties mainly in memory, particularly delayed verbal memory, but also in executive functioning. There is general accordance in considering executive dysfunctions as the “core” of the cognitive profile of young patients with anti-NMDAR encephalitis.

Research data support the hypothesis that the different and selective recovery of the cognitive functions may be related to the variable density and distribution of NMDA receptors in different areas of the brain. This may result in a different effect of immunotherapy on regions of the brain characterized by low and high NDMAR density, which may lead to a fast or slow recovery of the cognitive function controlled by these areas of brain. In particular, the NMDARs are highly concentrated in the frontal cortex and hippocampus, which explain the prevalence of deficits in memory and executive function in this disease [65]. It is still not clear if the variability of neuropsychological and psychological symptoms found in children and adolescents with anti-NMDAR encephalitis may be related to the developmental process of the specific frontal-subcortical circuits. NMDARs show age-related maturation and their concentration in the nervous system may depend on the stage of development [66]. It has been hypothesized that this disease may affect the development of the frontal subcortical circuits related with NMDA transmission [66].

Several prognostic factors of positive recovery, such as absence of tumor, absence of admission to an intensive care unit and early immunotherapy, have been identified [2,8,15,67]. Regarding the association between the type of treatment and the outcome of the disease, a consensus has not yet been reached [67]. Over the time, increasing data described the importance of second-line immunotherapy as an additional factor for clinical outcome [67]. In 2018, McKeon et al., through an evaluation based on the Rankin Scale, showed an association between second-line therapy and a favorable outcome [68], supporting the emerging evidence of case reports showing the positive influence of second-line therapy on cognitive skills. Despite this, early immunotherapy is currently considered to produce favorable cognitive outcomes [51,68]. In a recent study on adults, Wang et al. described an improvement in verbal memory abilities in patients with moderate-to-severe anti-NMDAR encephalitis who received early second-line therapy [69]. Regarding the pediatric age, data on the role of early treatment and second-line therapy are more scarce. To the best of our knowledge, no study described the effect of different treatments on neuropsychological skills in children/adolescents diagnosed with anti-NMDAR encephalitis. Moreover, given the great variability in treatment protocols and timing and neuropsychological tools used in previous research and case reports, results on the possible association between treatment and cognitive performance in children with anti NMDAR are difficult to generalize. Beyond treatment, another factor that may influence the prognosis of patients with anti-NMDAR encephalitis, including that concerning cognitive decline, is represented by the co-existence of anti-MOG (anti-myelin oligodendrocyte glycoprotein) [70,71,72]. Unfortunately, the search for the latter type of autoantibodies has become frequent only in recent years, thus data about the clinical picture of these patients are still incomplete. A limited recovery of cognitive functions in patients with anti-NMDAR encephalitis may also be secondary to a progressive cortical atrophy [73]. A quantitative volumetric analysis conducted in a recent study in both adult and pediatric patients, showed brain atrophy in the majority of patients (64%) [73].

Despite the emerging insights, the reviewed literature showed several limitations and heterogeneity in the survey methods used. Firstly, given the rarity of the disease, which has been characterized from a clinical and biochemical point of view only recently, most studies include only case reports or small samples [21,22,37,38,41,47]. It is also important to point out that the older studies concern clinical cases in which there was no strong consensus regarding the therapy of this disease, which unlike today was not systematized. This makes older results even less homogeneous than newer ones. Secondly, the timing of follow-up and the psychological and neuropsychological tools used for patients’ evaluation are extremely variable (Table 1). In some cases, a standardized investigation of the neuropsychological profile was not even performed [22,41,46]. Different studies might use different methods to comprehensively evaluate neuropsychological functions, such as intellectual functioning, memory, verbal fluency, visuomotor skills, attention and working memory. Lastly, a structured evaluation of pre-morbid intellectual and cognitive functioning is generally lacking (Table 1).

In order to understand and learn to manage the needs of patients with anti-NMDAR encephalitis, particularly regarding the impact this disease can have on daily life and school performance, additional neuropsychological research involving larger samples, longitudinal studies and more homogenous methods is required.

## 5. Conclusions

The present review article suggests that even several months after hospital discharge, most children and adolescents who have suffered from anti-NMDAR encephalitis may show neuropsychological sequelae involving memory, especially verbal memory, attentional resources and, in particular, executive functions. However, despite the following critical issues related to this disease, the emerging data suggest a favorable outcome from a long-term perspective. In fact, some cognitive skills, such as general intelligence, show good overall recovery over time. Additionally, although children diagnosed with anti-NMDAR encephalitis usually return to their daily life and previous activities, they may show a low quality of life.

Additional neuropsychological research evaluating larger samples, more homogenous methods and longitudinal studies is required.

## Figures and Tables

**Table 1:** Summary of the results issued from the available studies.

Author	Subjects Informations	Neuropsychological Domains Assessed (Tests Used)	Time of Assessment	Main Impaired Areas
[21]	1 patient: 8 years old, F	General intelligence abilities (WISC IV)	First evaluation: 15 months after symptoms onset	General intelligence (IQ = 78)
			Second evaluation: not performed	/
[31]	10 prospectivelly recruited patients Age range 3–17 years (at symptoms onset) F: 5; M: 5	-General intelligence abilities (GMDS 2–8; WPPSI III; WISC III and IV; WAIS-R; Leiter-R) -Receptive and expressive language: naming tests (BVN 5–11, BVN 12–18); verbal comprehension (Token Test for Children, 2nd edn; Test for Reception of Grammar); phonemic and semantic verbal fluency (BVN 5–11, BVN 12–18) -Selective and sustained attention (Barrage Task; Bell test and Coding—a Wechsler subtest) -Planning (Tower of London and Block Design—a Wechsler subtest) -Short-term verbal memory (Digit Span—a Wechsler subtest) and short-term visuo-spatial memory (Corsi’s Block Tapping Test Forward-BVN 5-11, BVN 12-18) -Visual-motor integration (Copying Form Test of Developmental Test of Visual-Motor Integration, and Rey–Osterrieth Complex Figure test)	First evaluation: a median of 3 months after symptoms onset (range 1–12 months)	General intelligence (IQ < 85 in 6 patients) Language: naming (4 patients); verbal comprehension (4 patient); Semantic verbal fluency (5 patients); Phonemic verbal fluency (4 patients) Attention: Sustained attention (3 children), selective attention (3 patients), conding (5 patients) Planning: below the normal range in 5 patients Memory: Short-term verbal memory (8 patients), Short-term visuo-spatial memory (2 patients) Visual-motor integration (5 patients)
			Second evaluation: a median of 27 months after symptoms onset (range 12–60 months)	Language: naming skills (1 patient), phonemic verbal fluency (2 patients) Attention: Sustained attention (2 children), selective attention (1 patient), conding (3 patients) Planning: below the normal range in 4 patients Memory: short-term verbal memory (1 patient), short-term visuo-spatial memory (1 patient)
	3 retrospectively recruited patients Age range: 7–13 years (at symptoms onset) F:3; M:0		One evaluation: range 31-112 months after symptoms onset	General intelligence (IQ < 85 in 1 patient) Language: phonemic verbal fluency 1 patient) Attention: Sustained attention (1 children), conding (2 patients) Planning: difficulties in 2 patients Memory: short-term verbal (2) and visuo-spatial memory (1 patient) Visual-motor integration difficulties in 2 patients
[37]	Patient 1: F Age at symptoms onset: 4 years Age of assessment: 4 years and 10 months Patient 2: M Age at symptoms onset: 3 years and 8 months Age of assessment: 5 years and 5 months	-General intelligence abilities (GMDS 2–8) -Receptive and expressive language (Test for Reception of Grammar—BVN 5–11; Peabody Picture Vocabulary Test; Naming Test—BVN 5–1; Fluency-5–11) -Selective and sustained attention (Bell Test) -Executive functions (Tower of London) -Visual and verbal memory (Corsi’s Block Tapping Test Forward; Digit Span Forward; Luria memory words test—BVN 5–11) -Visual-motor integration (Developmental Test of Visual-Motor Integration)	First evaluation Patients 1: 10 months after symptoms onset Patients 2: two years after symptoms onset	-IQ: 85 in patient 1 and 65 in patient 2 -Language: activation and integration of semantic information (significant deficit in the rapid naming test) -Attention: selective and prolonged -Problem-solving tasks and thinking flexibility -Verbal fluency (intrusions and perseverations at switching and clustering semantic tasks) -Spatial visuoconstructive abilities were impaired in patient 2
			Second evaluation Patient 1: Twenty-two months after symtoms onset Patient 2: not performed (he was followed-up at the same hospital where he was first admitted	In patient 1, the authors described a normalization of the IQ (96) and improvements in problem-solving tasks and in the selective/prolonged attention, clustering semantic tasks and switching.
[37]	Patient 1: 17 years old, F	-General intelligence abilities (WAIS-IV) -Language (Multilingual Aphasia Examination; Boston Naming Test; Delis–Kaplan Executive Function System verbal fluency) -Attention (Continuous Performance Test—II;) -Executive functions (Trail Making test; Tower of London; Delis–Kaplan Executive Function System- Trail making test; Delis–Kaplan Executive Function System—color–word interference; Symbol Digit Modalities—oral; stroop test; Rey–Osterrieth Complex Figure Test) -Memory (Boston naming test; California Verbal Learning Test—II; Wechsler Memory Scale—IV Logical Memory; WMS—IV Visual Reproduction; Peabody Picture Vocabulary Test; Hopkins Verbal Learning Test) -Motor/sensory (Grooved Pegboard) -Visual-spatial (Developmental Test of Visual Perception; Developmental Test of Visual-Motor Integration; Judgment of Line Orientation; clock drawing)	Three evaluations (4-24 months) Acute phase (4–6 weeks since symptoms onset) Post acute phase (2–6 months since symptoms onset) Outcome (6–24 months since symptoms onset)	Acute phase: attention; cognitive flexibility; visual-motor construction
				Post-acute phase: mild weaknesses in verbal memory and more substantial impairment in problem-solving and language (e.g., confrontational naming and comprehension of instructions)
				Outcome: problem-solving, confrontational naming, comprehension of instructions, and verbal recognition, were still below average (borderline to impaired ranges).
	Patient 2: 16 years old, F			Acute phase: she was very disoriented and appeared to be in a state of delirium
				Outcome: weaknesses in executive functioning, particularly inhibition and working memory, retrieval-based verbal memory, confrontational naming, and fine motor dexterity bilaterally (low average to borderline ranges).
	Patient 3: 18 years old, F			Acute phase: widespread and significant cognitive deficits across all assessed domains (borderline to severely impaired ranges)
				Post-acute phase: impairments in many areas of speech (e.g., comprehension, word finding, fluency, and/or prosody, neologisms); attention and thought processes were grossly impaired
				Outcome: weakness (low average to severely impaired ranges) in language skills (naming, repetition, fluency, and speeded reading) and mild but variable weakness with speeded responding
[31]	6 patients Age range (at evaluation): 6,11–13,6 years F: 4; M:2	-Mini mental state pediatric examination (acute phase, that precluded a full neuropsychological evaluation; before discharge) -General intelligence abilities/reasoning (Raven Colored Matrices; GMDS 2-8) -Language: naming and semantic fluency (BVN 5–11 and BVN 12–18) -Attention and speed: selective and sustained visual attention (Bell Test; Trial Making Test A) -Executive functions: phonemic fluency (BVN 5–11 and BVN 12–18); working memory (backward Digit Span Test—BVN 5–11 and BVN 12–18); frontal lobe functioning (Frontal Assessment Battery); shifting (Trial Making Test B) -Short-term verbal and visual-spatial memory memory (Digit Span Test and the Corsi’s Block Tapping Test—BVN 5–11 and BVN 12–18); verbal learning and long term verbal memory (word’s list and list recall—BVN 5–11 and BVN 12–18) -Visual motor integrations (Coding test of the WISC-IV; Rey–Osterrieth Complex Figure Test)	First evaluation: one month after discharge	Reasoning: below the normal range in 1 patient Attention (3/5 patients) Executive functions (5/5 children) Visual-motor abilities, implicating executive involvement (2/5 patients)
			Second evaluation: 35 months after discharge (range 24–48 months)	Attention (1/4 of the patients) Executive functions (2/4 patients) Visual-motor abilities in 2/4 children
	1 children: 15 months old, M		Four evaluations: from 1 month to 27 months after discharge	The child gradually deteriorated in all scales: Global score Locomotor Personal/social Eye/hand coordination Performance and, in particular Hearing/language
[40]	16 patients underwent a full neuropsychological evaluation Age range of disease onset: 3–17 years Age range at the assessment: 6–25 years F:15; M: 1	-Language: words comprehension and word findings (Boston Naming Test; Token Test) -Attention: reaction time, sustained attention, speed (CANTAB; Dutch Dot Cancellation Test—Bourdon–Vos) -Executive functioning: Intra-Extra Dimensional Set Shift, Spatial Span, Stockings of Cambridge (all CANTAB); Word Generation (NEPSY); Behavior Rating Inventory of Executive Function (BRIEF—Self-Report and BRIEF—Adult Questionnaire) -Visual and verbal memory (Paired Associated Learning (CANTAB), Rey Auditory Verbal Learning Test) -Quality of life: Pediatric Quality of Life Inventory (PedsQL Self-Report and PedsQL Parent Proxy-Report) -Fatigue: PedsQL Multidimensional Fatigue Scale questionnaire (PedsQL-MFS Self-Report and PedsQL-MFS Parent Proxy-Report)	One evaluation: a median of 31 months after symptoms onset (interquartile range 15–49, range 5–91)	Attention: sustained attention; speed Memory: long-term verbal and visual memory Language: naming High fatigue and low QoL Correlation between fatigue and QoL. No correlations between QoL, fatigue, sustained attention and long-term verbal memory

WISC III, Wechsler Intelligence Scale for Children-third edition; WISC IV, Wechsler Intelligence Scale for Children-fourth Edition; WPPSI III, Wechsler Preschool and Primary Scale of Intelligence—third edition; WAIS-R, Wechsler Adult Intelligence Scale—Revised; GMDS 2–8, Griffiths Mental Development Scales 2–8 years; Batteria di valutazione neuropsicologica per l’età evolutiva 5–11 years; Batteria di valutazione neuropsicologica per l’età evolutiva 12–18 years; CANTAB, Cambridge Neuropsychological Test Automated Battery; NEPSY, Developmental NEuroPSYchological Assessment Battery.
